# Assessment of the Concentration of Transforming Growth Factor Beta 1–3 in Degenerated Intervertebral Discs of the Lumbosacral Region of the Spine

**DOI:** 10.3390/cimb46110763

**Published:** 2024-11-11

**Authors:** Rafał Staszkiewicz, Dorian Gładysz, Dawid Sobański, Filip Bolechała, Edward Golec, Małgorzata Sobańska, Damian Strojny, Artur Turek, Beniamin Oskar Grabarek

**Affiliations:** 1 Collegium Medicum, WSB University, 41-300 Dabrowa Gornicza, Poland; drdsobanski@gmail.com (D.S.); sobanskamalgorzata05@gmail.com (M.S.); strojny.ds@gmail.com (D.S.); bgrabarek7@gmail.com (B.O.G.); 2 Department of Neurosurgery, 5th Military Clinical Hospital with the SP ZOZ Polyclinic in Krakow, 30-901 Cracow, Poland; gladyszdorian875@gmail.com; 3 Department of Neurosurgery, Faculty of Medicine in Zabrze, Academy of Silesia, 40-555 Katowice, Poland; 4 Department of Neurosurgery, Szpital sw. Rafala in Cracow, 30-693 Cracow, Poland; 5 Department of Forensic Medicine, Jagiellonian University Medical College, 31-531 Cracow, Poland; filip.bolechala@uj.edu.pl; 6 Department of Rehabilitation in Orthopaedics, Faculty of Motor Rehabilitation, Bronisław Czech University of Physical Education, 31-571 Krakow, Poland; bgolec@poczta.onet.pl; 7 Institute of Health Care, National Academy of Applied Sciences in Przemyśl, 37-700 Przemyśl, Poland; 8 New Medical Techniques Specjalist Hospital of St. Family in Rudna Mała, 36-060 Rzeszów, Poland; 9 Chair and Department of Biopharmacy, Faculty of Pharmaceutical Sciences in Sosnowiec, Medical University of Silesia, Katowice, 41-200 Sosnowiec, Poland; a.turek75@gmail.com; 10 Department of Molecular Biology, Gyncentrum, Laboratory of Molecular Biology and Virology, 40-851 Katowice, Poland

**Keywords:** intervertebral disc, intervertebral disc degeneration, transforming growth factor beta

## Abstract

The purpose of this study was to evaluate the feasibility of using the expression profile of transforming growth factor beta (TGF-β-1-3) to assess the progression of L/S spine degenerative disease. The study group consisted of 113 lumbosacral (L/S) intervertebral disc (IVD) degenerative disease patients from whom IVDs were collected during a microdiscectomy, whereas the control group consisted of 81 participants from whom IVDs were collected during a forensic autopsy or organ harvesting. Hematoxylin and eosin staining was performed to exclude degenerative changes in the IVDs collected from the control group. The molecular analysis consisted of reverse-transcription real-time quantitative polymerase chain reaction (RT-qPCR), an enzyme-linked immunosorbent assay (ELISA), Western blotting, and an immunohistochemical analysis (IHC). In degenerated IVDs, we noted an overexpression of all TGF-β-1-3 mRNA isoforms with the largest changes observed for TGF-β3 isoforms (fold change (FC) = 19.52 ± 2.87) and the smallest for TGF-β2 (FC = 2.26 ± 0.16). Changes in the transcriptional activity of TGF-β-1-3 were statistically significant (*p* < 0.05). Significantly higher concentrations of TGF-β1 (2797 ± 132 pg/mL vs. 276 ± 19 pg/mL; *p* < 0.05), TGF-β2 (1918 ± 176 pg/mL vs. 159 ± 17 pg/mL; *p* < 0.05), and TGF-β3 (2573 ± 102 pg/mL vs. 152 ± 11 pg/mL) were observed in degenerative IVDs compared with the control samples. Determining the concentration profiles of TGF-β1-3 appears to be a promising monitoring tool for the progression of degenerative disease as well as for evaluating its treatment or developing new treatment strategies with molecular targets.

## 1. Introduction

Degenerative disease of the intervertebral disc (IVD) of the lumbosacral (L/S) spine is a common spinal ailment that is affecting increasingly younger people [1,2,3]. It is estimated that 60–90% of the population will experience pain symptoms of the L/S spine as a result of degenerative disease at least once in their lifetime [1,2].

The IVD is divided into two main parts, the AF and the nucleus pulposus (NP) [4,5,6,7]. The determining factor for the occurrence of spinal pain over the course of the degenerative disease of IVD is the penetration of free nerve endings deep into the annulus fibrosus (AF) of the IVD from the surrounding environment [8,9,10,11,12]. Repeated damage to the connections between the vertebral endplates and the IVD leads to a reduction in hydration and the ability of nutrients to permeate into the IVD, creating conditions for neoinversion [8,9,10,11,12].

From a molecular aspect, in the etiology of the induction and development of pain complaints resulting from L/S spine IVD degenerative disease, it is indicated that a significant role is played by neurotrophic factors, which are primarily secreted by nervous system cells and immune system cells [13,14,15].

Among the transforming growth factor beta (TGF-β) superfamily proteins involved in the induction and progression of L/S spine IVD degenerative disease are the TGF-β family and the glial-cell-derived neurotrophic factor family [16,17,18,19]. Nonetheless, knowledge regarding the induction and development of degenerative spine disease on the molecular level, including the role of TGF-β superfamily molecules, remains fragmented [20]. Hence, TGF-β signalization is required for normal development and IVD growth and may be involved in IVD degeneration [20].

The NP typically exhibits a higher concentration of TGF-β due to its role in promoting extracellular matrix (ECM) production and especially high concentrations of proteoglycans and collagen type II, which are critical for the NP’s function as a load-bearing, compressive structure. TGF-β signaling in the NP supports matrix synthesis and cell survival, helping to maintain disc hydration and prevent degeneration. On the other hand, the AF has a lower concentration of TGF-β compared to the NP. This difference is consistent with the AF’s composition, which is more focused on producing collagen type I for tensile strength rather than proteoglycans for compression resistance. While TGF-β is still involved in ECM synthesis in the AF, particularly in response to injury or stress, its expression and activity are less prominent compared to that in the NP [4,5,6,7,21,22].

Chen et al. found that TGF-β expression changes with age and degeneration progression, though studies in human IVD models have yielded inconclusive results [20]. Nerlich et al. reported elevated TGF-β1 expression in degenerative IVDs with further studies linking TGF-β1 upregulation to degeneration severity [23,24,25]. Schroeder et al. observed decreased TGF-β in NP tissues but increased expression in AF tissues [26], while Abbott et al. found lower TbRI expression in severely degenerated NPCs [27]. Tsarouhas et al. noted no significant differences in TGF-β1 mRNA expression between herniated and control IVD tissues [28]. Tolonen et al. demonstrated increased TGF-β expression in osteoarthritic IVD samples, underscoring its role in spinal osteoarthritis [29]. Additionally, studies showed that TGF-β concentration rises with the radiological progression of IVD degeneration [30].

TGF-β1 accounts for over 90% of TGF-β activity and is crucial in various biological processes, including angiogenesis and ECM regulation in intervertebral disc (IVD) degeneration [20]. TGF-β1 promotes endothelial cell proliferation and adhesion, contributing to angiogenesis and inflammation during fibrosis [30,31]. TGF-β1 supports ECM synthesis and reduces apoptosis in early degeneration stages but also drives angiogenesis and fibrosis as degeneration progresses, particularly through VEGF expression in response to hypoxia [20,32,33,34,35].

Therefore, the aim of this study was to assess changes in the concentration profile of TGF-β-1-3 mRNA and proteins in degenerated IVDs of the lumbosacral (L/S) spine, depending on the degree of degeneration.

## 2. Materials and Methods

### 2.1. Ethical Considerations

This study was performed according to the 2013 Declaration of Helsinki guidelines on human experimentation. Data confidentiality and patient anonymity were maintained at all times. Patient-identifying information was deleted before the database was analyzed. Identifying patients individually in this article or in the database is impossible. Informed consent was obtained from all the patients. Approval from the bioethical committee operating at the Regional Medical Chamber in Krakow (No. 162/KBL/OIL/2021) was obtained for this study. The issue of obtaining post-mortem material for research is regulated by the Act of 1 July 2005, on the collection, storage, and transplantation of cells, tissues, and organs (Journal of Laws of 2020, Item 2134) [36].

Written informed consent was obtained from all the patients involved in the study, including consent to publish this paper. Each participant was fully informed of the nature of the study, and their rights were thoroughly protected throughout the process.

### 2.2. Study Group

The study group comprised 113 patients (55 women, 49%; 58 men, 51%; mean age of 45.5 ± 1.5 years) diagnosed with IVD disease of the L/S spine, who met the criteria for microdiscectomy surgery. The diagnosis process involved a comprehensive assessment, including magnetic resonance imaging (MRI), clinical symptom evaluation, neurological and physical examinations, and a detailed patient interview. Patients included in the study had IVD degeneration characterized by prolapse or extrusion and experienced persistent discogenic pain or symptomatic sciatica that had not improved after at least six weeks of non-surgical treatment. Due to the lack of symptom relief or worsening of their condition, these patients were scheduled for surgical intervention. This selection ensured that the study group represented individuals with confirmed, isolated degeneration of the L/S spine IVD, which was consistent with criteria established for surgical intervention. Specific criteria for the inclusion and exclusion of patients in the study group were presented in previous publications [37,38]. The study applied specific inclusion and exclusion criteria to ensure a consistent sample within the study group of patients with isolated intervertebral disc (IVD) degeneration in the lumbosacral spine. Eligible participants were adults over 18 years old who demonstrated lumbosacral spine IVD degeneration characterized by disc prolapse or extrusion on MRI. Inclusion also required patients to have experienced discogenic pain or symptomatic sciatica that had not responded to non-surgical treatment for at least six weeks. Additionally, participants had to have no other coexisting spinal pathologies, and the disease duration needed to be between six and twelve weeks to align with the study’s focus on recent, isolated degeneration cases. Conversely, patients under 18 years old were excluded, as were those with MRI findings of protrusion or sequestration-type disc degeneration. Individuals with prior surgical interventions for lumbosacral spine degeneration, spine-related inflammatory or autoimmune diseases, or a history of trauma were also excluded. Other exclusion criteria for the study group included mental disorders such as dementia, polyneuropathy, pregnancy, and the presence of significant coexisting diseases, various types of cancers (e.g., metastatic tumors, leukemia, and spinal cord tumors), osteoporosis, and active spinal infections. Patients with less than six weeks or more than twelve weeks of disease duration were also excluded to ensure uniformity in the timeframe of disease progression under investigation [37,38].

The degree of advancement of the L/S spine IVD degenerative disease was defined using the Pfirrmann scale [22]. Pfirrmann developed a five-stage radiological classification system to evaluate the severity of IVD degeneration using MRI [39,40,41].

In 27 patients, the advancement of degenerative changes corresponded to grade 2 on the Pfirrmann scale; 43 patients had grade 3 advancement of changes according to the Pfirrmann scale; in 32 patients, the changes corresponded to grade 4 according to the Pfirrmann scale; and in 11 patients, the changes corresponded to grade 5 on the Pfirrmann scale. Specifically, 18 IVDs (15.93%) were taken from the L1/L2 segment, 26 cases (23%) from the L2/L3 segment, 27 cases (23.89%) from the L3/L4 segment, 32 cases (28.32%) from the L4/L5 segment, and 10 cases (8.86%) from the L5/S1 segment [38].

### 2.3. Control Group

The control group consisted of 81 participants (43 women, 53%; 38 men, 47%; a mean age of 31.5 ± 1.5 years old) from whom L/S spine IVD samples were collected post-mortem during a forensic autopsy or organ collection no more than 48 h after death. IVDs were removed from the L1/L2 segment in 12 cases (14.81%), the L2/L3 segment in 16 cases (19.75%), the L3/L4 segment in 18 cases (22.22%), the L4/L5 segment in 27 cases (33.33%), and the L5/S1 segment in 8 cases (9.89%) [38].

Specific criteria for the inclusion and exclusion of patients in the control group were presented in previous publications. The control group participants were selected based on specific inclusion and exclusion criteria to ensure a comparison group without signs of IVDs degeneration. Participants in the control group were required to be up to 45 years of age and show no signs of degeneration in the collected tissue samples, as confirmed by microscopic examination with hematoxylin and eosin staining (H&E). Additionally, eligible participants had no history of neoplastic diseases or inflammatory conditions affecting the spine or surrounding tissues. Participants were excluded from the control group if they were over 45 years old or if microscopic examination revealed any degenerative features in the collected material. A history of neoplastic disease or inflammatory spinal conditions also led to exclusion, including conditions such as osteomyelitis, IVD inflammation, epidural empyema, shingles, arthritis, inflammatory infiltrates of the rectum, Scheuermann’s disease, and Paget’s disease. Those with a general history of spinal or inflammatory disease were not eligible for the control group, ensuring that participants represented a non-degenerated baseline [37,38].

The confirmation of the absence of degenerative changes in the collected IVD samples was performed using H&E staining in accordance with the study protocol. An example of H&E staining is shown in our previous publication [38].

### 2.4. Securing the Extracted Material for Molecular Examination

The clinical materials (IVDs) were extracted from patients in the study group during a microdiscectomy using the en bloc method as well as from deceased patients. After the IVDs were thoroughly washed to remove blood, they were placed into sterile Eppendorf tubes pre-filled with TRIzol reagent (Invitrogen Life Technologies, Carlsbad, CA, USA) and stored at −80 °C until the molecular section of the experiment commenced (RT-qPCR, ELISA, Western blot analysis) or embedded in paraffin (POL-AURA, Dywity, Poland) for the immunohistochemical (IHC) analysis. Storing samples at low temperatures effectively minimizes endogenous RNase activity, which is a critical precaution for ensuring successful RNA extraction [42,43,44]. The tissue samples were generally small and consistent with the needs of histological and biochemical analyses. The typical size of the samples ranged from [insert specific dimensions, e.g., 1–2 cm in diameter and 0.5 cm in thickness], allowing for both immediate study and long-term storage.

The molecular analysis was performed on 113 degenerated IVD samples and 81 control samples.

### 2.5. Extraction of Whole Ribonucleic Acid (RNA)

First, the study and control samples were homogenized using a hand-held homogenizer (T18 Digital Ultra-Turrax, IKA Polska Sp. z o.o., Warsaw, Poland) until no solid fragments were visible [42,44,45].

The extraction of whole RNA from the study and control samples was conducted using a modified Chomczyński–Sacchi method with the use of TRIzol reagent (Invitrogen Life Technologies, Carlsbad, CA, USA), as recommended by the manufacturer. After this procedure, the extracts were dried and stored in this form at a temperature of −80 °C until the next stage of the molecular analysis. An assessment of the RNA extract quality was conducted using electrophoretic separation in a 1% agarose gel stained with ethidium bromide at a concentration of 0.5 mg/mL (SigmaAldrich, St. Louis, MO, USA), and a quantitative assessment was performed through a spectrophotometric measurement (Nanodrop^®^, Thermo Fisher Scientific, Waltham, MA, USA).

### 2.6. Determination of Changes in the Expression Profile of TGF-β-1-3 mRNA in Degenerated and Control IVDs Using RT-qPCR

The RT-qPCR reaction was performed using a set of Sensi-Fast™ One-Step Probe Assay reagents (Bioline, London, UK). RT-qPCR was conducted in 50 µL of reaction mixture with the following thermal profile: reverse transcription (45 °C, 10 min); polymerase activation (95 °C, 2 min); and 40 three-step cycles consisting of denaturation (95 °C, 5 s), hybridization (60 °C, 10 s), and annealing (72 °C, 5 s). The RT-qPCR primers were purchased from Genomed (Gdańsk, Poland), and their nucleotide sequence is provided in Table 1. Glyceraldehyde 3-phosphate dehydrogenase (GAPDH) was used as an endogenous control for RT-qPCR. For each biological repeat, three technical repeats were conducted.

Because the PCR efficiency ranged from 90% to 110% and GAPDH had stable expression across all samples (both healthy and diseased), changes in gene expression were presented as the normalized relative mRNA expression (2^−∆∆Ct^ method), where 1 corresponded to an equal expression of the given gene in the study and control samples; a result below 1 corresponded to the decreased expression of the gene in the study samples compared with the control; and a result above 1 indicated an overexpression of the given gene in the study samples compared to the control.

### 2.7. Determination of the Profile of TGF-β-1-3 Proteins Through ELISA and Western Blot Separation Procedures in Degenerated and Healthy IVDs

Changes in the concentration profile of TGF-β-1-3 in normal and degenerated IVDs were determined using ELISA and electrophoretic separation in a polyacrylamide gel (Western blot), using the following antibodies: polyclonal anti-TGF-β1 antibody bs-0086R (STI, Poznan, Poland; 1:1000 dilution), polyclonal anti-TGF-β2 antibody bs-20412R (STI, Poznan, Poland; 1:1000 dilution), and polyclonal anti-TGF-β3 antibody bs-0099R (STI, Poznan, Poland; 1:1000 dilution), according to the manufacturer’s recommendations.

GAPDH sc-47724 (GAPDH; Santa Cruz Biotech, Dallas, TX, USA; 1:500 dilution) was used as the endogenous control protein. The secondary antibody used was HRP-conjugated goat anti-rabbit IgG (BioRad, Milan, Italy; catalog number 1706515; 1:3000 dilution). The absorbance at 540 nm was measured using an M200PRO plate reader (Tecan, Männedorf, Switzerland).

A detailed protocol for conducting the ELISA and Western blot procedures is provided in previous papers [37,38].

### 2.8. IHC Analysis

The specimens were sectioned on a microtome (Leica Microsystems, Wetzlar, Germany) into 8.0 µm thick serial slices. The subsequent preparation steps of the tissue sections, such as dehydration, antigen retrieval, antibody incubations, and staining, were performed according to the manufacturer’s instruction manuals (DAB Substrate Kit, Peroxidase (HRP), Vector Laboratories, Newark, California, USA, and IHC-Paraffin Protocol (IHC-P), Abcam plc, Cambridge, UK). The obtained immunohistochemical reactions were examined and captured on a Nikon Coolpix fluorescent optic system. Both the cellular location of the selected proteins and their quantity were assessed through a computer image analysis using the ImageJ software [46]. A total of 15 photographs were taken from three slides of each patient under 200× magnification. Using the ImageJ software with the IHC-Profiler plug-in [47], the optical density of the DAB reaction products was evaluated in the fields where the immunohistochemical reaction occurred in response to the presence of the selected proteins. An average percentage of the DAB-stained area was also calculated in relation to the background values in each field. An example of H&E staining is shown in our previous work [38].

### 2.9. *Statistical Analysis*

The statistical analysis of the results was performed by assuming a statistical significance threshold (*p*) of <0.05 in Statistica 13 PL software (Statsoft, Krakow, Poland). The Shapiro–Wilk test was used to assess the conformity of the data distribution with a normal distribution, which was confirmed. Accordingly, the next statistical analysis stage used parametric methods, i.e., Student’s *t*-test for independent groups or a one-way ANOVA and Tukey’s post hoc test. The homogeneity of variance was checked using Levene’s test.

## 3. Results

### 3.1. Changes in the Expression Profile of TGF-β-1-3 mRNA in Degenerated and Control IVDs

In degenerated IVDs, we noted an overexpression of all TGF-β-1-3 mRNA isoforms with the largest changes observed for *TGF-β3* isoforms (fold change (FC) = 19.52 ± 2.87) and the smallest for TGF-β2 (FC = 2.26 ± 0.16). Changes in the transcriptional activity of TGF-β-1-3 were statistically significant (*p* < 0.05).

### 3.2. TGF-β-1-3 Protein Expression Profile Determined Through the Enzyme-Linked Immunosorbent Assay (ELISA) Technique

The expression levels of the TGF-β1, TGF-β2, and TGF-β3 isoforms were assessed in both the study group (patients with degenerated IVDs) and the control group (participants with non-degenerated IVDs) using the ELISA technique. The results revealed significantly elevated concentrations of all three TGF-β isoforms in the degenerated IVDs when compared to the control group (Table 2; *p* < 0.05).

In the study group, TGF-β1 and TGF-β3 were expressed at comparable levels, while TGF-β2 exhibited the lowest concentration among the three isoforms. On the other hand, in the control group, TGF-β1 was the most abundantly expressed isoform with TGF-β3 being the least expressed (Table 2; *p* < 0.05).

A further analysis of the study group, stratified according to the severity of disc degeneration using the Pfirrmann grading system (grades 2 to 5), revealed a dynamic pattern in the concentration of the TGF-β isoforms. The concentrations of all three isoforms progressively increased with the advancement of degenerative changes from Pfirrmann grade 2 through grade 4 (Table 2; *p* < 0.05). Specifically, the concentration of TGF-β1 increased from 2876 ± 123 pg/mL in grades 2 and 3 to 198 ± 176 pg/mL in grade 4. Similarly, TGF-β2 increased from 1834 ± 201 pg/mL in grade 2 to 2043 ± 156 pg/mL in grade 4, and TGF-β3 increased from 2545 ± 165 pg/mL in grade 2 to 2761 ± 209 pg/mL in grade 4 (Table 2).

However, in the most advanced stage of degeneration (Pfirrmann grade 5), a decline in the concentrations of all three TGF-β isoforms was observed. The TGF-β1 levels decreased to 1987 ± 156 pg/mL, the TGF-β2 levels decreased to 1761 ± 198 pg/mL, and the TGF-β3 levels decreased to 2219 ± 187 pg/mL (Table 2; *p* < 0.05). These findings suggest that while the TGF-β expression is initially upregulated in response to disc degeneration, there may be a threshold at which further degeneration results in a decrease in these cytokine levels.

### 3.3. TGF-β-1-3 Concentration Profile in Degenerated and Control IVDs Determined Using the Western Blot Technique

**Figure 1:**
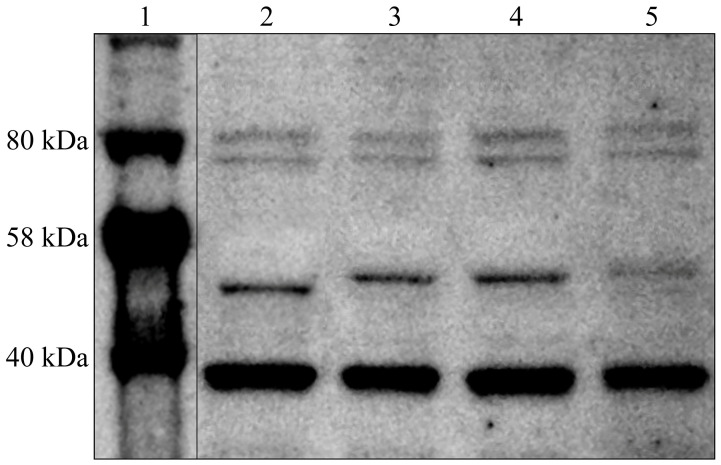
Normalized expression of TGF-β-1-3 in IVDs normalized against GAPDH expression. Track 1, molecular weight marker; Track 2, TGF-β-1;Track 3, TGF-β-2; Track 4;, TGF-β-3, Track 5, GAPDH.

The expression profile of TGF-β-1-3 in degenerated and control IVDs, determined using the Western blot technique, was the same as that noted in the ELISA procedure. Figure 1 presents an example electropherogram that confirms the specificity of the reaction as well as the nativity of the samples (based on the GAPDH result; molecular mass of 36 kDa). Normalized relative to GAPDH, the band optical density for TGF-β1 (molecular weight of 44 kDa) in the study samples was 9.87 ± 4.31, and it was 0.67 ± 0.12 in the control samples (*p* < 0.05). Normalized relative to GAPDH, the band optical density for TGF-β2 (molecular weight of 50 kDa) in the study samples was 3.29 ± 0.98, and it was 0.91 ± 0.28 in the control samples (*p* < 0.05). Normalized relative to GAPDH, the band optical density for TGF-β3 (molecular weight of 47 kDa) in the study samples was 5.98 ± 1.09, and in the control samples, it was 0.18 ± 0.07 (*p* < 0.05).

**Figure 2:**
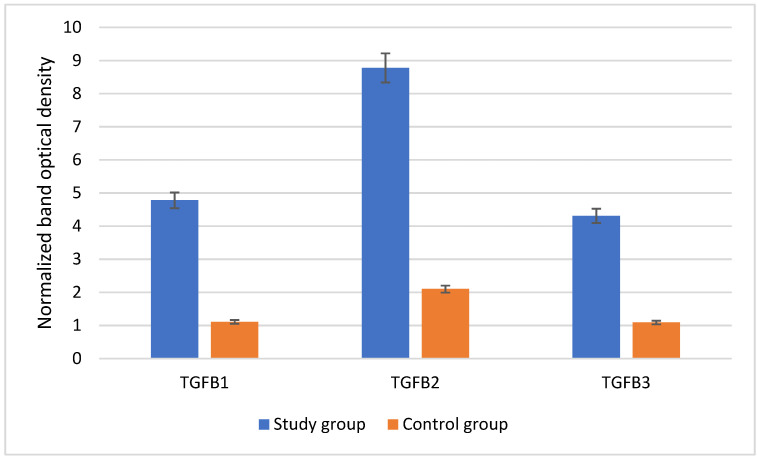
Band optical density of TGF-β-1-3 in L/S spine IVDs collected from the study and control groups determined using Western blotting. TGF-β-1-3, transforming growth factor beta isoforms.

The normalized band optical density of TGF-β-1-3 in degenerated and control IVDs is presented in Figure 2.

### 3.4. TGF-β-1-3 Expression in IVDs Collected from Patients in the Study Group and Control Group Participants, Determined Through the IHC Technique

The expression of TGF-β1, TGF-β2, and TGF-β3 was visualized in both degenerated and non-degenerated (control) intervertebral discs (IVDs) using the IHC technique.

Quantitatively, the optical density of the IHC reaction product revealed a significantly higher expression of the TGF-β isoforms in the degenerated IVDs compared to the control group. For TGF-β1, the optical density in the degenerated IVDs was 430.63% of the control group (*p* < 0.05), showing a marked upregulation in degenerated tissues (Table 3). Similarly, the optical densities for TGF-β2 and TGF-β3 in the study group were 418.10% and 395.41% of the control, respectively (*p* < 0.05), reflecting substantial increases in these isoforms in degenerated IVDs as well.

The study group also showed variations in TGF-β expression across different stages of degeneration, as classified by the Pfirrmann grading system. For TGF-β1, the optical density increased progressively from Pfirrmann grade 2 (3.29 ± 0.91) to grade 4 (7.29 ± 1.87) before decreasing in grade 5 (2.09 ± 0.54). A similar trend was observed for TGF-β2, with the highest optical density observed in Pfirrmann grade 4 (12.11 ± 2.32), which was followed by a decrease in grade 5 (10.57 ± 1.98). TGF-β3 also exhibited progressive increases from Pfirrmann grade 2 (2.00 ± 0.14) to grade 5 (5.68 ± 0.34), though this pattern was less pronounced than for TGF-β1 and TGF-β2 (Table 3).

**Figure 3:**
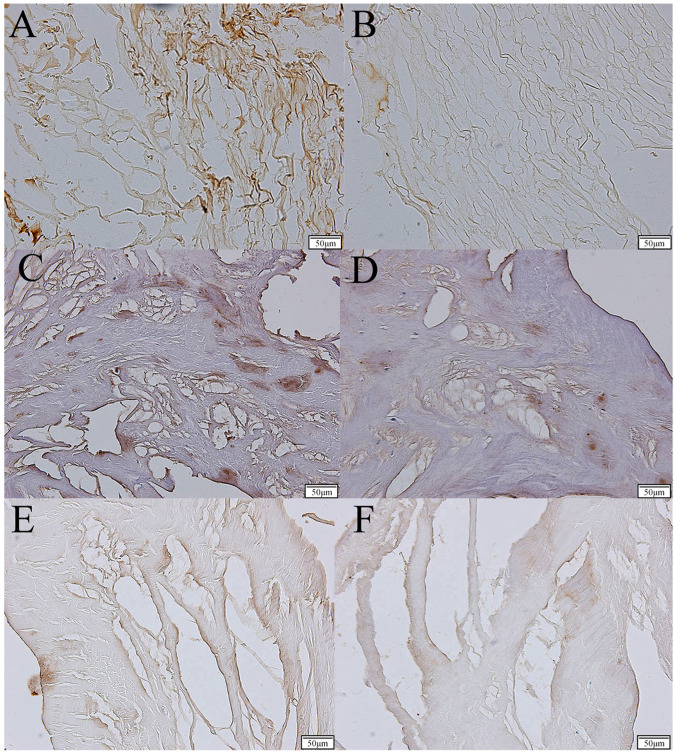
The immunochemical expression of TGF-β-1-3 in the study and control samples. TGF-β-1-3, transforming growth factor beta 1-3. (**A**)—expression of TGF-β-1 in the study group; (**B**)—expression of TGF-β-1 in the control group; (**C**)—expression of TGF-β-2 in the study group; (**D**)—expression of TGF-β-2 in the control group; (**E**)—expression of TGF-β-3 in the study group; (**F**)—expression of TGF-β-3 in the control group.

These findings suggest that the expression of TGF-β isoforms is significantly upregulated in degenerated discs and varies according to the stage of degeneration. The highest expression levels are generally observed in mid-level degenerative stages (Pfirrmann 3 and 4), followed by a decline in the most advanced degenerative stage (Pfirrmann 5), indicating a potential complex role for TGF-β in the pathophysiology of disc degeneration. Examples of the immunochemical expression of TGF-β-1-3 in the study and control samples are presented in Figure 3.

## 4. Discussion

The TGF-β family plays a very important role in homeostatic behavior over the course of spinal osteoarthritis [48,49,50]. In mammals, three TGF-β isoforms have been identified, namely TGF-β1, TGF-β2, and TGF-β3 [51].

It seems that TGF-β fulfills a protective function in the development of IVD degenerative disease through the activation of extracellular matrix synthesis or through the inhibition of catabolic processes occurring in the cell [52]. Nonetheless, the overexpression of TGF-β family members is considered a promoting factor for IVD degenerative changes, namely by contributing to an increase in the nerve growth factor (NGF) concentration, which is considered a significant factor in the etiopathogenesis of pain over the course of IVD degeneration [20,53]. It seems that the duality of TGF-β’s action may be related to the pathways activated by this cytokine [49,54]. In joints not affected by the degenerative process, the TGF-β expressed by chondrocytes exerts a protective effect through the SMAD2/3 pathway, whereas during the degenerative process, the SMAD1/5/8 pathway is activated [55]. Bian et al. [52,56] noted in a mouse model of IVD degeneration that the administration of a TGF-βR1 receptor inhibitor inhibited the signalization of R-SMAD, contributing to a decrease in the grade of IVD degeneration [52,56]. However, Kwon et al. [57] noted an increase in the concentration of TGF-β1 and the activation of the SMAD2/3 and SMAD1/5/8 pathways in bovine IVDs, whereby the SMAD1/5/8 cascade may contribute to the inhibition of the SMAD2/3 pathway, the result of which was an advancement in IVD degeneration [57]. Nevertheless, it should be noted that the vast majority of the studies regarding the role of TGF-β in IVD degenerative disease were conducted on animal models and lasted a few days or weeks, whereas in the case of human tissues and cells, there exists a tendency for aging and the degeneration of material. Therefore, further analyses are necessary [49].

Previous studies have indicated that the concentration of some pro-inflammatory cytokines, such as tumor necrosis factor (TNF) and interleukin (IL)-1β, increases over the course of the IVD degeneration process, promoting the degradation of the ECM. This is mediated by an increased expression of catabolic enzymes, including matrix metalloproteinases (MMPs) and a disintegrin and metalloproteinase with thrombospondin motifs (ADAMTS) [58,59]. Therefore, it is suggested that TGF-β may exert a regulatory effect on the expression of genes encoding proteins responsible for ECM degradation [60,61,62,63]. On the other hand, observations conducted regarding signalization dependent on TGF-β indicate that it may exert not only a destructive but also a protective effect on the ECM [60,61,62,63].

It seems that TGF-β actively participates in the synthesis of glycosaminoglycans (GAG) through the SMAD-dependent pathway, the Ras homolog gene family member A (RHOA)/Rho-associated protein kinase (ROCK) pathway, and the mitogen-activated protein kinase (MAPK) pathway [64,65,66,67,68]. Furthermore, TGF-β induces the expression of aggrecan, proteoglycans, and type II collagen [64,65,66,67,68]. This confirms the duality of TGF-β’s action and the signaling cascades activated by it, while the biological effect exerted by TGF-β is dependent on age [64,65,66,67,68].

In vivo research regarding the expression pattern of TGF-β in the context of IVD degenerative disease was conducted on both animal and human models.

Nagano et al. [69] determined the expression of TGF-β-1-3 and its receptors (TGF-βRI-III) through immunohistochemical staining using IVDs obtained from 10 mice at the ages of 8, 24, and 50 weeks after birth as a research model [69]. These authors demonstrated the expression of TGF-β-1-3 and TGF-βRI-III in 8-week-old mice, noting a decrease in the concentration together with the age of the mice. This observation confirmed the involvement of TGF-β-1-3 in postnatal IVD development and the degeneration process [69].

The same observations as those of Nagano et al. [54] were noted in a study conducted by Matsunaga et al. [70]. Additionally, Zhang et al. [71] determined a decrease in the SMAD2 and SMAD3 protein concentrations in C57BL/6 mice at 2 and 18 months old [71].

In this article, the expression profile of TGF-β-1-3 was determined in the whole IVD, not separately in the NP and AF. Therefore, a valuable addition to this study would be to determine the TGF-β-1-3 expression in the NP and AF. However, during the collection of IVDs from study group participants, the en bloc technique was used, which makes it difficult to separate the two compartments of the IVD structure. This technique involves the removal of a herniated disc in one single, intact piece (en bloc) rather than in fragments, as is typical in a traditional microdiscectomy. As a result, the anatomical boundaries between the AF, NP, and CEP may be preserved as they are extracted together, potentially complicating the post-operative separation of these components for further study or analysis.

In turn, Murakami et al. [72] indicated an increased expression of TGF-β1 mRNA in the NP and AF of rabbits at 6 months and 3 years of age [72]. Moreover, Hiyama et al. [73] determined the overexpression of TGF-β2, TGF-β3, SMAD3, and SMAD5 in rat NPs, while SMAD3 expression decreased in the AF [73]. Changes in the expression profile of TGF-β-1-3 at the MRNA and protein levels in an animal model suggest that its expression is not only dependent on the IVD compartment but can also be regulated by microRNA (miRNA) [74,75].

The significant costs, pain, and disability associated with degenerative disc disease (DDD) highlight the critical need for a biological agent capable of mitigating the progression of this condition [76].

Additionally, reports indicating the use of TGF-β-dependent signaling as a potential target in IVD degenerative disease therapy, using molecular targets, are worthy of attention. Yang et al. indicated an overexpression of TGF-β1 in a coculture model of NP cells with bone marrow mesenchymal cells compared to cultures containing NP cells alone, which was associated with the inhibition of nuclear factor kappa B (NF-κB)-dependent signaling [32,33]. Additionally, Xiong et al. [77] compared the concentration of TGF-β1 and proteins dependent on signalization activated by this cytokine in cervical spine IVDs in a group of patients with cervical osteoarthritis in relation to the co-occurrence of osteophytes or ossifications around the IVD [77]. Additionally, emerging treatments for IVD degeneration involve the use of platelet-rich plasma (PRP), which contains a platelet-derived growth factor, TGF-β, an epidermal growth factor, insulin-like growth factor 1, and VEGF. These components exhibit potent anti-inflammatory and regenerative properties that promote the repair of damaged tissue [78]. A 2016 prospective randomized controlled trial enrolled 47 patients to evaluate the PRP treatment for IDD. Of these, 29 received PRP injections, while the remaining 18 were administered only contrast agents. The group treated with PRP injections demonstrated pain relief and improved functionality as early as eight weeks post-treatment with benefits persisting for at least one year and no reported adverse events [79].

In vivo experiments have demonstrated that the transfection of TGF-β1 in rabbit NP cells led to increased proteoglycan synthesis [80]. Similarly, the transfection of TGF-β1 in aged human NP cells promoted the production of both proteoglycans and collagen [22]. In turn, Matta et al. found a decrease in the expression of TGF-β1, connective tissue growth factor (CTGF), and WNT1-inducible signaling pathway protein 2 (WISP2) in degenerative disc NPs, suggesting a link to the loss of notochordal cells (NCs). Notably, in their pre-clinical models, treatment with a combination of TGF-β1 and CTGF successfully restored a healthy, cellular NP rich in NCs and stem cells compared to the sham control discs. Furthermore, their findings showed that the TGF-β1 and CTGF treatment enhanced cell viability, deoxyribonucleic acid (DNA) synthesis, and the expression of healthy ECM genes in NP cells derived from degenerative human discs [81]. In their review report, Hodgkinson et al. also evaluated the therapeutic potential of TGF-β in IVD degeneration. They found that GDF (100 ng/mL) with TGF-β1 had a synergic effect on promotor chondrogenic differentiation [82]. Previous research has identified that a single injection of a combination of recombinant human (rh) TGF-β1 and CTGF proteins into the NP of an injured intervertebral disc (IVD) can effectively mediate IVD degeneration in pre-clinical rodent models. In this study, a novel molecular therapy, NTG-101, containing rhTGF-β1 and rhCTGF proteins suspended in an excipient solution was developed, and its efficacy was evaluated using in vivo models of DDD, including the rat-tail model and chondrodystrophic (CD) canines. The findings demonstrated that a single intra-discal injection of NTG-101 produced anti-degenerative effects, leading to a reduction in the expression of pro-inflammatory cytokines such as IL-1β, IL-6, and IL-8, as well as ECM-degrading enzymes such as MMP-13 and cyclooxygenase-2 (Cox-2). Furthermore, the treatment induced pro-anabolic effects in the IVD-NP, restoring the expression of healthy extracellular matrix (ECM) proteins, including aggrecan and collagen 2A1 [83]. In turn, Risbud et al. demonstrated that TGF-β3 can enhance the structure and function of the NP and AF by increasing the levels of activated ERK1/2, which in turn regulates TGF-β receptors I and II (TGF-β-RI and TGF-β-RII) [84]. Hegewald et al. confirmed this finding and suggested that the administration of TGF-β3 could be a potential candidate for the biological treatment of AF degeneration. They showed that AF stimulation with TGF-β3 was associated with an increased production of type X collagen [85]. Given the results proposed for the potential use of TGF-β in IVD degeneration [22,79,80,81,82,83,84], along with the dual nature of TGF-β’s role in the disease [31,86,87], it appears that administering TGF-β within the IVD is justified in the early and late stages of degenerative disc disease (Pfirrmann grades 2 and 5). However, greater therapeutic success in treating IVD degeneration would likely be observed in the early stages of degeneration. On the other hand, in the intermediate stages of IVD degeneration, it seems that molecular treatments should focus on inhibiting the expression of TGF-β and its related pathways. Nevertheless, further research is necessary.

The safety concerns surrounding gene therapy pose challenges for its clinical use. Specifically, the potential risk of tumor formation associated with high-dose exposure and prolonged application in treating chronic IVD degeneration has emerged as a significant issue. Advancements in the reliability of viral vector designs and better control over transgene expression could pave the way for safer and more effective clinical applications of gene therapy [88,89].

The current study, while providing valuable insights into the role of TGF-β isoforms in IVDs degeneration, has several limitations that future research could address to deepen understanding. Notably, investigating the methylation status of TGF-β-1-3 genes in both degenerated and healthy disc tissues could reveal epigenetic regulation in response to degeneration, potentially indicating reversible pathways. Additionally, miRNAs may modulate TGF-β-1-3 expression in disc tissues with specific miRNAs potentially acting as regulators or biomarkers for early-stage degeneration. Employing a microarray analysis could expand understanding by identifying co-expressed genes and pathways that interact with TGF-β, including those involved in inflammation and extracellular matrix degradation. Moreover, assessing the prevalence of polymorphic variants within TGF-β1-3 genes may reveal genetic predispositions, guiding personalized interventions targeting specific isoforms. Finally, next-generation sequencing (NGS) could provide a more comprehensive view of the genomic landscape, identifying novel mutations, rare variants, and alternative splicing events affecting TGF-β and related pathways.

For similar studies, it is important to consider limitations in sample inclusion criteria. Cases with coexisting spinal diseases, history of spine-related inflammatory or neoplastic diseases, or those with past spinal surgeries should generally be excluded to maintain focus on isolated degeneration. Similarly, age restrictions may be necessary, as degeneration rates and TGF-β expression vary with age, potentially impacting results. Including or excluding these groups can influence findings, so future studies should tailor selection criteria based on specific study aims, ensuring uniformity and reducing confounding factors.

## 5. Conclusions

This study underscores the central role of TGF-β signaling in L/S IVDs degeneration with TGF-β-1 emerging as the most impactful isoform. Notably, all TGF-β isoforms (TGF-β-1, TGF-β-2, and TGF-β-3) were overexpressed during the early stages of degeneration, which was likely as a response to shifting microenvironmental conditions within the discs. As degeneration advanced from grades 2 to 4 on the Pfirrmann scale, the concentrations of these isoforms steadily rose, indicating an active role of TGF-β signaling in the progression of degeneration. However, in the most advanced degeneration stage (grade 5), the isoform levels dropped, suggesting that TGF-β’s influence wanes as degeneration becomes more severe, highlighting its potential as a therapeutic target during earlier stages. Comparative findings from prior studies suggest that the effects of TGF-β are shaped by patient age, species, detection methods, and tissue type—factors that future research should consider refining therapeutic strategies. These results advocate for an in-depth exploration of TGF-β signaling, including its epigenetic regulation via methylation, miRNA interactions, and genetic variations. Advanced tools such as NGS could further illuminate the genetic landscape of TGF-β in IVD degeneration, offering a foundation for precise diagnostic markers and targeted therapies.

## Figures and Tables

**Table 1:** Nucleotide sequence of the primers used in RT-qPCR for TGF-β-1-3 and GAPDH.

mRNA	Oligonucleotide Sequence	Tm (°C)
TGF-β1	Forward: 5’-GGCCAGATCCTGTCCAAGC-3’ Reverse: 5’-GTGGGTTTCCACCATTAGCAC-3’	85.4
TGF-β2	Forward: 5’-CAGCACACTCGATATGGACCA-3’ Reverse: 5’-CCTCGGGCTCAGGATAGTCT-3’	88.7
TGF-β3	Forward: 5’-CTGGATTGTGGTTCCATGCA-3’ Reverse: 5’-TCCCCGAATGCCTCACAT-3’	86.6
GAPDH	Forward: 5’-GGTGAAGGTCGGAGTCAACGGA-3’ Reverse: 5’-GAGGGATCTCGCTCCTGGAAGA-3’	86.4

Forward, sense primer; reverse, antisense primer; Tm, melting temperature; GAPDH, glyceraldehyde 3-phosphate dehydrogenase; TGF-β-1-3, transforming growth factor beta 1-3. The specificity of RT-qPCR was confirmed by determining the melting temperature for each amplimer.

**Table 2:** Concentration of TGF-β-1-3 in L/S spine IVDs collected from the study and control groups, determined using ELISA.

Isoform of TGF-β	Group	Concentration (pg/mL)	95% Cl
TGF-β1 ^a,b,d,e^	Control	276 ± 19	210–345
	Study	2797 ± 132	2541–3098
	Pfirrmann 2	2876 ± 123	2456–3087
	Pfirrmann 3	3127 ± 165	2987–3456
	Pfirrmann 4	3198 ± 176	2898–3298
	Pfirrmann 5	1987 ± 156	1765–2321
TGF-β2 ^a,b,e^	Control	159 ± 17	140–198
	Study	1918 ± 176	1561–2198
	Pfirrmann 2	1834 ± 201	1656–2098
	Pfirrmann 3	2034 ± 165	1871–2198
	Pfirrmann 4	2043 ± 156	1876–2198
	Pfirrmann 5	1761 ± 198	1456–2001
TGF-β3 ^a,b,c,d,e^	Control	152 ± 11	134–189
	Study	2573 ± 102	2345–2761
	Pfirrmann 2	2545 ± 165	2456–2671
	Pfirrmann 3	2767 ± 187	2571–2981
	Pfirrmann 4	2761 ± 209	2671–2891
	Pfirrmann 5	2219 ± 187	2091–2348

TGF-β-1-3, transforming growth factor beta isoforms; 95% CI, 95% confidence interval; a, statistically significant difference in protein concentration between study and control groups (*p* < 0.05); b, statistically significant difference in protein concentration between Pfirrmann 4 and Pfirrmann 5 groups (*p* < 0.05); c, statistically significant difference in protein concentration between Pfirrmann 2 and Pfirrmann 4 groups (*p* < 0.05); d, statistically significant difference in protein concentration between Pfirrmann 2 and Pfirrmann 5 groups (*p* < 0.05); e, statistically significant difference in protein concentration between Pfirrmann 3 and Pfirrmann 5 groups (*p* < 0.05). Results are presented as mean ± standard deviation.

**Table 3:** The optical density of the reaction product for selected proteins in IVDs obtained from the study and control groups.

Isoform of TGF-β	Group	Optical Density	95% Cl
TGF-β1 ^a,b,c,e,f^	Control	1.11 ± 0.13	1.04–1.18
	Study	4.78 ± 0.65	4.45–5.11
	Pfirrmann 2	3.29 ± 0.91	2.83–3.75
	Pfirrmann 3	6.42 ± 1.23	5.79–7.04
	Pfirrmann 4	7.29 ± 1.87	6.34–8.24
	Pfirrmann 5	2.09 ± 0.54	1.82–2.36
TGF-β2 ^a,c,f^	Control	2.10 ± 0.32	1.93–2.26
	Study	8.78 ± 0.18	8.69–8.87
	Pfirrmann 2	5.44 ± 0.23	5.33–5.56
	Pfirrmann 3	7.01 ± 0.55	6.73–7.28
	Pfirrmann 4	12.11 ± 2.32	10.94–13.28
	Pfirrmann 5	10.57 ± 1.98	9.57–11.57
TGF-β3 ^a,c,d,e,f^	Control	1.09 ± 0.18	0.99–1.18
	Study	4.31 ± 0.98	3.81–4.81
	Pfirrmann 2	2.00 ± 0.14	1.93–2.07
	Pfirrmann 3	4.34 ± 0.71	3.98–4.70
	Pfirrmann 4	5.21 ± 0.56	4.93–5.49
	Pfirrmann 5	5.68 ± 0.34	5.51–5.85

TGF-β-1-3, transforming growth factor beta 1-3. Data are presented as mean ± standard deviation. a, statistically significant difference in protein concentration between study and control groups (*p* < 0.05); b, statistically significant difference in protein concentration between Pfirrmann 4 and Pfirrmann 5 groups (*p* < 0.05); c, statistically significant difference in protein concentration between Pfirrmann 2 and Pfirrmann 4 groups (*p* < 0.05); d, statistically significant difference in protein concentration between Pfirrmann 2 and Pfirrmann 5 groups (*p* < 0.05); e, statistically significant difference in protein concentration between Pfirrmann 3 and Pfirrmann 5 groups (*p* < 0.05). Results are presented as mean ± standard deviation. f, statistically significant difference in protein concentration between Pfirrmann 3 and Pfirrmann 4 groups (*p* < 0.05).

## Data Availability

The data used to support the findings of this study are included in the article. The data cannot be shared due to third-party rights and commercial confidentiality.
